# Supplementary material for: Performance during the Glittre-ADL test between patients with and without post-tuberculosis bronchiectasis: A cross-sectional study
Source: PLoS One. 2023 Sep 1;18(9):e0290850. doi: 10.1371/journal.pone.0290850 (PMC10473510; doi:10.1371/journal.pone.0290850)
Supplement: Attachment — Submitted filename: Review.pdf [file pone.0290850.s002.pdf]

Review/Comments:

This is a cross-sectional study that proposed to evaluate the performance of Post-tuberculosis bronchiectasis patients during TGlittre by comparing them with nonposttuberculosis bronchiectasis patients and to evaluate the determinants of performance during TGlittre. In addition, this patients completed Short Form-36, handgrip strength, quadriceps muscle strength and pulmonary function tests.
The article presents some corrections and adjustments to be made, in addition to some doubts about the study methods.
The comments are presented in an attached text.

Title:

- As followed by the authors, STROBE suggests that the type of study be included in the title.

Abstract:

- Check the correct spelling of Post Tuberculosis
- "and evaluate the determinants of lung performance during TGlittre."- In the text of the article, lung performance is not mentioned. Please change the abstract.
- Conclusion: see comments on the conclusion in the text.

Introduction:

- "In Brazil, the incidence of TB was 31.6 cases/100,000 people in 2020, placing the country among those with the highest TB burden [7]." – Authors should review this citation. This information is from another article cited in reference article 7. This data is presented in the introduction of reference 7, following another reference.

- "...this test has never been used previously in PTBB patients."- I suggest that the authors change this sentence to "there are no reports in the scientific literature of the use of this test".

Methods:

- How did the authors calculate the sample size? I also suggest that the authors present a flowchart with the study design and sample characterization.

- How was the recruitment of these patients?

- I suggest making explicit how the clinical diagnosis of bronchiectasis is performed.

- "or clinical evidence of TB"- what clinical evidence?

- Were patients with COPD or asthma associated with bronchiectasis excluded?

- Exclusion criteria: I suggest adding individuals with a neurological history or diagnosis.

- Were the patients using any medication that would influence test performance, such as bronchodilators?

- The acronym CAAE (Protocol of Approval in the Ethics Committee) means Certificate of Presentation of Ethical Appreciation, which indicates that the project was sent to the Ethics Committee, but does not mean approval. I suggest showing the approval number.

- Did the authors use the FACED, BSI or other document to assess the severity of bronchiectasis in these patients?

- The authors used the SF-36 to assess the quality of life in patients with bronchiectasis. Why was this questionnaire used? Are there other questionnaires that could be used, more specific for this population?

- "Maximum strength was assessed after a 5-s sustained contraction of the dominant leg, and the highest value of three attempts with 1-min intervals was considered for analysis

[20].”- I suggest reviewing the reference used to perform this test. Reference 20 is from an article on functional capacity in women with rheumatoid arthritis.

- “the variability of the TGlitre logarithmic time”. – I kindly ask you to correct the name of the test.

Results:

- Table 2 – In this table, was any data presented in mean ± SD? If not, kindly remove it from the legend.

- In some tables, the results presented were in median and others in mean and standard deviation. Why did the authors recommend presenting most of the results in median and not in mean?

Discussion:

- “It is estimated that years of life lost account for approximately one-quarter, whereas years lived with disability account for three-quarters (77%) of the total burden of disease associated with post-TB pulmonary impairment [16].”- I apologize, as I did not understand this sentence presented in the discussion.

- “We observed that PTBB and NPTBB patients had poor performance on TGlittre, although they did not differ significantly from each other.”- This is the main objective of the study and the authors did not discuss it. Why do you think there was no significant difference? And clinical difference?

- “In these patients, the longer the TGlittre time was, the worse the lung function, muscle function and QoL.”- Wouldn't it be the other way around?

- “In addition, lung volume and QMS are the determinants of TGlittre performance in PTBB patients, whereas in NPTBB patients, the determinants are respiratory muscle strength, degree of air trapping and QMS”. – Respiratory muscle or expiratory muscle?

- “To the best of our knowledge, this is the first study to evaluate the determinants of TGlittre in PTBB patients compared to those in NPTBB patients. “-But the authors did not evaluate the determinants in comparison, they are individual determinants for that specific group.

- “In the present study, we observed that PTBB patients took 52% longer to perform the TGlittre tasks compared to NPTBB patients, although the groups did not differ significantly.”- It was not 52% compared to the other group, but in relation to the predicted value, described in the article by Reis et al. (ref 26), as described by the authors on page 13.

- “...to methodological differences in the performance of TGlittre.”- What would those differences be?

- “In our sample of NCFB patients, obstructive damage was the most common finding and can be explained by several mechanisms, such as mucosal edema, presence of secretions, collapse of the airways due to the greater malleability of the affected bronchi, and concomitant infections [9,31].”- I apologize, but I did not understand the reason for references 9 and 31 to be in this sentence, since the authors are commenting on their results and not comparing or discussing with the scientific literature.

- “Peripheral muscle strength is a strong determinant of functional capacity to exercise in chronic lung diseases [34].”- Is this reference correct? The article is about elderly and hand grip only. And the mean/median age of the groups were not elderly.

Conclusion:

- “In conclusion, our study shows that PTBB patients have poor performance on TGlittre, which is similar to that of NPTBB patients.” - What would be poor performance? Wouldn't it be better below expected? The two groups were lower than expected, but it is worth mentioning the main objective of the study, informing that there was no difference between the 2 groups.

- The performance on TGlittre in PTBB patients is largely explained by lung volume and QMS, while in NPTBB patients, it is largely explained by respiratory muscle strength, degree of air trapping and QMS. – What would be largely explained? I suggest changing it to explained. The correct is respiratory muscle strength or expiratory muscle strength?

References:

- 40.5% are articles from the last 5 years.
