# Supplementary material for: Performance during the Glittre-ADL test between patients with and without post-tuberculosis bronchiectasis: A cross-sectional study
Source: PLoS One. 2023 Sep 1;18(9):e0290850. doi: 10.1371/journal.pone.0290850 (PMC10473510; doi:10.1371/journal.pone.0290850)
Supplement: Attachment — Submitted filename: PONE-D-23-17193_REVIEW.pdf [file pone.0290850.s003.pdf]

Performance during the Glittre-ADL test: A comparative study between patients with and without posttuberculosis bronchiectasis (PONE-D-23-17193)

This well-designed cross-sectional study provides valuable insights into the functional capacity of patients with posttuberculosis bronchiectasis and non-posttuberculosis bronchiectasis using the TGlittre test. The study lays a foundation for developing effective interventions and personalized care for individuals burdened by these chronic lung diseases by unravelling key determinants of functional performance. The study's significance lies in its contributions to bronchiectasis research and its potential to impact clinical practice and improve patients' quality of life. I have minor comments.

Introduction

Replace the sentence "Quality of life (QoL) impairment in patients with bronchiectasis is equivalent to the impairment observed in patients with severe chronic obstructive pulmonary disease (COPD)" by "The level of Quality of Life (QoL) impairment in patients with bronchiectasis is comparable to that observed in patients with severe chronic obstructive pulmonary disease (COPD)."

Replace the sentence "In the presence of bronchiectasis, peripheral muscle resistance and general fatigue seem to be significantly impaired [10]" by "Patients with bronchiectasis seem to exhibit impairment in peripheral muscle resistance and experience considerable general fatigue [10]."

Replace the sentence "The impact of posttuberculosis bronchiectasis (PTBB) on the lungs and muscles is sparsely described in the existing literature, and there is limited knowledge about its relationship with other forms of non-cystic fibrosis bronchiectasis (NCFB) [7,15]" by "The impact of posttuberculosis bronchiectasis (PTBB) on the lungs and muscles is sparsely described in the existing literature, and there is limited knowledge about its relationship with other forms of non-cystic fibrosis bronchiectasis (NCFB) [7,15]."

Methods

QOL: "…validated for the Brazilian Portuguese language."

Make it clear that each domain in the SF-36 Quality of Life (QOL) questionnaire has a maximum score of 100, and there is no total score for the entire SF-36 questionnaire.

Was the measurement of quadriceps strength performed isometrically? If yes, please refer to it as 'maximum quadriceps isometric contraction strength'.

Check the units (kg or kgf?). In "Results" the values are reported in kgf for QMS and Handgrip.

Results

Do not repeat the results in text and tables.

Discussion/Conclusion

The sentence "Our findings may provide information for outcome measures in pulmonary rehabilitation programs in patients with PTBB" should be moved to the Discussion. Additionally, I believe that your findings have the potential to offer valuable insights not only for outcome measures but also for proposing interventions in randomized controlled trials (RCTs).
